# The impact of tobacco control policies on smoking initiation in eleven European countries

**DOI:** 10.1007/s10198-019-01090-x

**Published:** 2019-08-09

**Authors:** Ali Palali, Jan C. van Ours

**Affiliations:** 1 Booking.com, Amsterdam, The Netherlands; 2 Erasmus School of Economics, Erasmus University Rotterdam, Rotterdam, The Netherlands; 3 Department of Economics, University of Melbourne, Parkville, Australia; 4 Tinbergen Institute, Rotterdam, The Netherlands; 5 CEPR, London, UK

**Keywords:** Tobacco control policies, Smoking initiation, Hazard rate models, I12, C41

## Abstract

We investigate the effect of tobacco control policies on smoking initiation in eleven European countries. Based on individual data about age of onset of smoking, we use hazard rate models to study smoking initiation. Thus, we are able to take into account observed and unobserved personal characteristics as well as the effect of the introduction of a variety of tobacco control policies including price and and non-price policies, i.e., bans on tobacco advertisements, smoke-free air regulation, health warnings on packages of cigarettes, and treatment programs to help smokers quit smoking. We find that higher tobacco prices have a negative effect on the initiation into smoking for males but not for females. We find no effect of non-price tobacco control policies on smoking initiation.

**Electronic supplementary material:**

The online version of this article (10.1007/s10198-019-01090-x) contains supplementary material, which is available to authorized users.

## Introduction

According to the World Health Organization tobacco use is an epidemic. In the past decades, a large amount of evidence has piled up about the adverse health consequences of tobacco use [24]. These health effects have recently reached an alarming peak, which makes tobacco use one of the biggest health threats ever, killing approximately one person every 6 s [29]. Nearly, 6 million people die every year because of tobacco-related diseases, a number which is expected to reach 8 million by 2030.

The gloomy health effects have stimulated many governments to introduce tobacco control policies. First and foremost, smoking is discouraged by imposing substantial taxes on tobacco products thus increasing prices. Furthermore, there are non-price tobacco control policies ranging from prohibition or restriction of advertisements of tobacco products to laws necessitating the placement of health warnings on tobacco product packages and from different types of anti-smoking campaigns to laws prohibiting the use of tobacco in certain places. Nearly, one-third of the world’s population is covered by at least one type of comprehensive non-price tobacco control policy and a considerable amount of resources is spent to enforce these policies [46].

To what extent tobacco control policies actually affect smoking is an empirical question. Past research on the effects of tobacco control policies is not conclusive. Whereas tobacco prices, bans on cigarette advertisements and placement of health warnings on tobacco and cigarette packages seem to have had a negative effect on the number of smokers and the per capita cigarette consumption, this is less clear for smoking bans, i.e., restrictions on smoking in public places and workplaces. Some studies find a negative effect on smoking, while others find no effect.

Most studies focus on the extensive or intensive margin of smoking, i.e., on the share of smokers in a population or the tobacco consumption per smoker. From a policy point of view, it is also important to assess the effects of tobacco control policies on smoking initiation. Early initiation has important effects later on in life. Van Ours [43] shows that the age of onset of tobacco use is an important predictor of life-time tobacco use. An individual who starts smoking at an early age has a higher probability of long-term tobacco use compared to someone who starts later. Sharapova et al. [41] find that earlier ages of tobacco use initiation among US middle and high school students are associated with sustained tobacco use and greater nicotine dependence. The authors conclude from this that the reinforcement of comprehensive efforts to reduce tobacco use initiation among youth is important to reduce overall youth tobacco use. Since the age of onset is a very strong predictor of addiction and quit behavior, analyzing the effects on smoking initiation is important not only for early smoking behavior, but also for smoking dynamics in general. Early ages of onset imply a greater probability of persistent smoking later on in life with serious health consequences. Pirie et al. [38], for example, show smoking to have a big effect on mortality rates of UK women. Of the female smokers, 53% died before age 80 years, while for never-smokers, this was 22%. There appeared to be a lifespan difference between smokers and non-smokers of 11 years. Pirie et al. [38] also find that stopping to smoke before age 40  years avoids more than 90% of the excess mortality. This also implies that early age of onset of smoking may have a big impact on mortality rates. The implicit assumption behind policies targeting youth’s smoking is that reducing smoking initiation at a young age reduces lifetime smoking propensities [19] and thus has positive health effects.

We analyze Eurobarometer data from Austria, Germany, Finland, France, Ireland, Italy, The Netherlands, Portugal, Spain, Sweden, and United Kingdom to study the effects of tobacco control policies on smoking initiation. We consider the effect of tobacco prices but also study the effects of non-price tobacco control policies in particular smoke-free air laws, bans on advertising promotion and sponsorship, health warnings on tobacco product packaging and treatment programs to help dependent smokers to stop smoking. The eleven European countries differ in the timing of introduction of the various tobacco control policies. To analyze the effects of these policies on smoking initiation, we use mixed proportional hazard rate models, which control for observed as well as unobserved determinants of smoking initiation.

Our study has three contributions to the literature on the effects of tobacco control policies. First, we contribute to the small literature about the effects of tobacco control policies on smoking initiation. Second, we present one of the first studies using individual level cross-country data, capturing the variation in the implementation of various tobacco control policies in European countries. The cross-country variation enables us to study the causal effects of tobacco control policies in an international context. Third, we use hazard rate models in our empirical analysis. The dynamics in smoking behavior are rather complex. Individuals start smoking over only a limited age range. If they have not started smoking by their early twenties, they are very unlikely to start smoking later on. Using a hazard rate framework allows us to take these peculiarities into account.

The setup for the remainder of our paper is as follows. In “Previous studies”, we provide an overview of the previous empirical studies on the impact of tobacco control policies. We distinguish between studies focusing on the extensive and intensive margin of smoking and studies focusing on smoking dynamics. “A simple model of smoking initiation” provides a simple theoretical model to explain how tobacco control policies may affect smoking initiation. “Smoking in Europe” presents information about tobacco control policies in Europe and discusses the data we use in the empirical analysis. These are from one survey, where we introduce a time dimension using information on the age of onset. “Empirical analysis” presents the setup of our analysis and our parameter estimates. In recent decades smoking of females has increased a lot, such that in some countries, smoking prevalence of females is not very different from the smoking prevalence of males. Nevertheless, in most countries, there is still a clear gender difference in smoking prevalence. Therefore, we do the analysis separately for females and males. Our main findings are that high tobacco prices reduce the onset of smoking for males, but not for females, while non-price tobacco control policies do not influence smoking initiation. Conclusions concludes.

## Previous studies

Tobacco advertisements aim to persuade youngsters to start smoking, encourage current smokers to keep smoking, and stimulate past smokers to restart smoking. They also aim to increase amounts of cigarettes smoked [45]. Cigarette advertisements can influence public discussions about negative consequences of smoking causing people to think that negative effects are overrated [9]. In short, tobacco advertisements encourage smoking and thus bans on these advertisements aim to remove this encouragement. Bans on advertisements are an important component of tobacco control policies. Early studies on the relationship between tobacco control policies and smoking are based on aggregate data using cross-country time series variation. Saffer and Chaloupka [40], for example, analyze the effects of banning tobacco advertisements on per capita cigarette consumption in 22 OECD countries over the period 1970–1992. They find that comprehensive laws prohibiting advertisements have strong effects on per capita cigarette consumption, whereas non-comprehensive laws have limited effects. Blecher [4] analyzes the effects of laws prohibiting cigarette advertisements in 52 countries worldwide over the period 1990–2005 finding that both comprehensive bans and limited bans have a significant negative impact on per capita cigarette consumption.

Other tobacco control policies are the placement of health warnings on tobacco and cigarette packages and general campaigns about the negative health consequences of smoking. Health warnings can affect smoking both at the intensive and the extensive margin, i.e., the number of cigarettes per smoker and the share of smokers in the population. Warnings convey direct information about adverse health effects of smoking. This may prevent young individuals from starting to smoke. Moreover, health warnings on cigarette packages may reduce the intensity of smoking or stimulate quitting from smoking.

Restrictions on smoking in public places or workplaces can directly affect cigarette consumption by making it harder for people to smoke. Moreover, such restrictions can easily affect the perception of smoking. First, these restrictions might work as a reminder of the negative consequences of smoking, especially for young individuals. Second, young individuals are less likely to be exposed to passive smoking thanks to these restrictions. The absence of smokers can affect their perception of cigarette consumption, i.e., when youngsters are less often exposed to smokers they may experience this as confirmation of negative health consequences of smoking.

In Table 1, we present a summary overview of studies that are mostly based on individual data. The effects of tobacco prices and tobacco control policies are usually based on calendar time variation, cross-state or cross-region variation or a combination of both. We distinguish between studies that focus on prevalence and intensity of smoking and studies that focus on smoking dynamics, i.e., smoking initiation and quitting.

**Table 1:** Overview of the previous studies on tobacco control policies

Study	Data		Time	Dependent variable	TCP	Effects
*a. Prevalence and intensity of smoking*						
Chaloupka and Grossman [8]	I	US	1992–1994	Youth smoking	Anti-smoking campaigns	Negative
Meier and Licari [31]	S	US	1955–1994	Cigarette consumption	Health warnings	Negative
Chaloupka and Wechsler [10]	I	US	1993–1996	Youth smoking	Smoking restrictions	Negative
Powell et al. [39]	I	US	1996	Tobacco use	Various TCP	Negative
					Tobacco prices	Negative
Adda and Cornaglia [2]	S	US	1970–2007	Per capita consumption	Various smoking bans	Mixed
					Cigarette prices	Negative
Nagelhout et al. [33]	I	NL	2001–2008	Smoking prevalence	Workplace smoking bans	Negative
					Bans bars and restaurants	No
Anger et al. [3]	I	DE	2002–2008	Smoking intensity	Smoke-free air	No
Jones et al. [21]	I	UK	1991–2007	Smoking prevalence	Public smoking bans	No
Boes et al. [5]	I	CH	2005–2011	Smoking prevalence	Public smoking bans	Negative
Villar and Nicolás [44]	I	ES	2006–2012	Smoking prevalence	Bans public venues	Negative
Cotti et al. [11]	I	US	2004–2012	Tobacco expenditures	Taxes	Negative
					Smoke-free air	No
DelBono et al. [15]	I	IT	1999–2005	Smoking behavior	Public smoking ban	No
Meier et al. [30]	I	CH	2001–2016	Smoking prevalence	Ban youth tobacco sales	Small negative
*b. Smoking dynamics*						
Douglas and Hariharan [17]	I	US	1978–1997	Initiation	Tobacco prices	No
Douglas [16]	I	US	1987	Initiation and quits	Tobacco prices	No
					Smoking restrictions	Positive (quits)
Forster and Jones [18]	I	UK	1984	Initiation and quits	Tobacco prices	Negative (initiation)
						Positive (quits)
López Nicolás [27]	I	ES	1993–1997	Initiation and quits	Tobacco prices	Positive
					Various bans	Mixed
DeCicca et al. [13]	I	US	1988–1992	Initiation	Tobacco prices	No
Kidd and Hopkins [23]	I	AU	1990–1998	Initiation and quits	Tobacco prices	Negative (initiation)
						No (quits)
Cawley et al. [7]	I	US	1990–2000	Initiation	Tobacco prices	Negative (males)
						No (females)
DeCicca et al. [14]	I	US	1988–2000	Initiation and quits	Tobacco prices	No (initiation)
						Positive (quits)
Nonnemaker and Farrelly [35]	I	US	1997–2006	Initiation	Tobacco prices	Negative
Lillard et al. [25]	I	US	1986–2007	Initiation	Tobacco prices	Negative
Marti [28]	I	CH	2007	Initiation and quits	TCP expenditures	Negative (initiation)
						Positive (quits)
Guindon [20]	I	VN	2003–2004	Initiation	Tobacco prices	Negative
Lillard and Önder [26]	I	TR	2008	Initiation and quits	Health warnings	Mixed (initiation)
						Positive (quits)

Data: *I* individual/household, *S* state/region averages

Country codes: *AU* Australia, *CH* Switzerland, *DE* Germany, *ES* Spain, *IT* Italy, *NL* Netherlands, *TR* Turkey, *UK* United Kingdom, *US* United States, *VN* Vietnam

### Prevalence and intensity of smoking

Chaloupka and Grossman [8] state that anti-smoking campaigns have had significant negative effects on youth smoking at both the intensive and the extensive margin. Using US data Meier and Licari [31] analyze the effects of health warnings on smoking along with the effects of taxes. They find that health warnings on cigarette packages have small but significant negative effects on cigarette consumption. Chaloupka and Wechsler [10] find that in the US, smoking restrictions have negative effects on smoking at both the intensive margin and the extensive margin. They also find that age restrictions on the access to tobacco seem to have had little impact as the effectiveness of such laws depends on their enforcement. Powell et al. [39] investigating smoking behavior of US high school students find that both cigarette prices and state-level and school-level tobacco control policies have a negative effect on tobacco use. These negative effects are reinforced through the existence of peer effects. Adda and Cornaglia [2] find that laws prohibiting smoking at workplaces do not have any effect on smoking prevalence or per capita cigarette consumption. However, they find that smoking bans in bars and restaurants have small but significant negative effects. Nagelhout et al. [33] find that the workplace smoking ban introduced in The Netherlands in 2004 decreased smoking prevalence, while the smoking ban in bars and restaurant introduced in 2008 did not have an effect. Anger et al.'s [3] study the effects of smoking bans who were gradually introduced in all of Germany’s federal states. Using a difference-in-differences approach, they find that the smoke-free legislation on average did not affect smoking behavior. However, among visitors of bars and restaurants, smoking and smoking intensity were reduced. Smoke-free legislation is primary aimed to protect non-smokers from the harm of second-hand smoking. However, this legislation may also induce smokers to quit smoking.^1^

Smoke-free legislation is not necessarily beneficial for non-smokers. Possibly, smoking bans in public places lead to more smoking at home. Jones et al.'s [21] study the effects of public smoking bans on smoking behavior exploiting the differential timing of the introduction of these bans in Scotland and England finding that they had limited short-run effects on both smoking prevalence and the total level of smoking. Boes et al.'s [5] study the effect of a Swiss smoking ban in public venues. Because these bans were introduced in different regions at different moments in time, they are able to use a difference-in-differences approach finding a negative effect on smoking rates 1 year after the implementation of the bans. Villar and Nicolás [44] analyze the effects of the Spanish clean air law finding a reduction in the proportion of households containing smokers. In a recent study, Cotti et al. [11] investigate the impacts of tobacco control policies finding that an increase in the tobacco taxes significantly reduced the tobacco consumption and increased the consumption of smoking cessation products. However, they also find that a smoke-free-air policy banning smoking in bars do not have any significant effects on the consumption of tobacco products. DelBono and Vuri [15] provide an overview of European studies on the effects of smoking bans on smoking behavior concluding that the evidence is mixed. They revisit the effects of the 2005 smoking ban in Italy showing that the previous studies that focused on a before–after comparison overestimate the effects of the ban because of not taking seasonal differences in smoking behavior into account. From a difference-in-difference setup, it appears that the smoking ban had no impact on smoking behavior. Meier et al. [30] investigate the effects of a ban on the sales of tobacco to teens that was gradually introduced across Switzerland (and EU countries). They find that this ban caused a less than 1% point reduction in teen smoking attributing this small effect to teens circumventing the bans through peers.

### Smoking dynamics

In addition to studies on prevalence and intensity of smoking, there are studies that focus on smoking dynamics. These studies are often based on cross-sectional data exploiting retrospective information about age of onset of smoking and the duration of smoking. DeCicca et al. [13] argue that their are clear differences between young and adults in the way prices affect smoking behavior. Since the onset of smoking is limited to early ages, for young smokers the main decision is whether or not to start smoking. For adult smokers, the decision is about intensity of smoking and about whether or not to quit smoking altogether. For youngsters, higher tobacco prices will reduce smoking mainly by preventing them to start smoking, while for adult smoking, higher tobacco prices will lead to cut down of quit smoking. According to Lillard et al. [25], models of smoking initiation based on longitudinal data are more relevant to policy analysis than models of the prevalence of smoking at a particular moment in time, because the decision to start smoking is different from the decision to continue smoking. Dynamics in smoking behavior have been studied using hazard rate models of smoking initiation sometimes in combination with hazard rate models of quitting smoking. Initially, the studies on smoking dynamics focused on the effect of cigarette prices only.

Panel b of Table 1 presents an overview of studies on smoking dynamics. Douglas and Hariharan [17] analyzing US data find that cigarette prices have no impact on smoking initiation. Douglas [16] analyzing US data finds no evidence of cigarette prices affecting smoking initiation, while quit rates increase with cigarette prices. Douglas [16] is among the few studies investigating the effects of non-price tobacco control policies on the dynamics in smoking.^2^ He uses a state-specific smoking restrictiveness index and finds evidence that a more restrictive policy promotes quitting from smoking but does not deter the decision to start smoking. Forster and Jones [18] analyze British data finding that tobacco prices have a small negative effect on smoking initiation, while they have a more substantial positive effect on quit rates from smoking. López Nicolás [27] analyzes Spanish data to establish the price sensitivity of smoking dynamics finding that prices have a very small effect on the propensity to start smoking, while an increase in the prices of the cheapest varieties of cigarettes encourages quitting from smoking. López Nicolás [27] also finds that the ban on smoking ads and smoking bans introduced in some public transport media in 1984 did not affect smoking dynamics, whereas the extension of the smoking bans to flights and intensified health warning campaigns a few years later seem to have had an effect on both starting and quitting. DeCicca et al. [13] find no effects of tobacco prices on the onset of smoking among US youngsters. Kidd and Hopkins [23] analyzing Australian data find that tobacco prices affect the onset of smoking but not the quit rate. Based on an analysis of US data, Cawley et al. [7] conclude that smoking initiation among males is negatively affected by cigarette prices, while these have no impact on smoking initiation among females. DeCicca et al. [14] also find no effect of tobacco prices on the onset of smoking although this study does find that higher tobacco prices are associated with increase quitting from smoking. Nonnemaker and Farrelly [35] find significant albeit small effects of tobacco prices on smoking initiation. Lillard et al. [25] argue that other studies may not have found an effect of prices on smoking initiation because of limited policy variation over the calendar time periods studied. They themselves use three datasets to allow for more calendar time variation in policies finding negative and significant price effects on smoking initiation. Marti [28] estimates the dynamics of smoking in Switzerland using tobacco control spending as one of the explanatory variables and finding that these affect both smoking initiation and quitting from smoking. Guindon [20] studies the impact of tobacco prices on the onset of smoking in Vietnam finding significant and substantial effects. Lillard and Önder [26] investigate the effects of information about health risks of smoking in Turkey finding that as new information arrives all smokers are more likely to quit smoking, while female non-smokers are less likely to start.

## A simple model of smoking initiation

A model of smoking initiation has to take into account that individuals start smoking in their teens or early twenties. If an individual has not started smoking in this age range, smoking initiation later on in life is very unlikely [43]. This implies that over a relatively short age range individuals balance marginal costs and marginal benefits of smoking initiation. Apparently, from a certain age onward, this balance is negative, i.e., individuals will refrain from smoking. Or, alternatively, individuals are no longer facing the balancing question as they already made up their mind that for the rest of their life it is better to abstain from smoking. It is also possible that for some individuals the costs of starting to smoke always outweighs potential benefits, i.e., they realize from early on in life that there are no utility gains in smoking initiation. And, it is also possible that later on in life individuals are no longer confronted with invitations to start smoking, because most or perhaps, all of their friends, family, and colleagues are non-smokers.

We present a simple model of smoking initiation with the purpose of illustrating how tobacco control policies may affect smoking initiation. We assume that individuals are confronted with a flow of smoking opportunities.^3^ The arrival rate of smoking opportunities is likely to be age-dependent. According to Suranovic et al. [42], individuals in their early teens expect no benefits of smoking and, therefore, have no interest in starting to smoke. If friends start smoking and encourage participation, youngsters may start to think that smoking has potential benefits. Furthermore, teenagers may experience peer pressure or follow the example of their parents. Conditional on having an opportunity to start smoking an individual will balance marginal benefits and marginal costs of doing so.

According to Douglas and Hariharan [17] a rational individual will start smoking if and only if the marginal benefit of the first cigarette is larger than its marginal cost:

1 $$\begin{aligned} {\mathrm{MB}}_t\left( C_t, Y_t, L_t \right) > {\mathrm{MC}}_t\left( C_t, Y_t, L_t \right) \end{aligned}$$

where *C* represents the consumption of a cigarette, *Y* is the consumption of other goods, *L* represents other life cycle events that affect utility, and *t* represents time.^4^ Both marginal cost and marginal benefits may be influenced by variables that have a stochastic component. Therefore, Eq. (1) can be divided into a non-stochastic and a stochastic component:

2 $$\begin{aligned} {\mathrm{MB}}_t^{*} + \varepsilon _t > {\mathrm{MC}}_t^{*} + \mu _t \end{aligned}$$

where ${\mathrm{MB}}_t^{*}$ and ${\mathrm{MC}}_t^{*}$ are the expected marginal benefits and costs of smoking. The probability of smoking initiation at time *t* can be written as

3 $$\begin{aligned} \begin{aligned} Pr(C_t>0)&=Pr({\mathrm{MB}}_t^* + \varepsilon _t> {\mathrm{MC}}_t^* + \mu _t) \\&=Pr({\mathrm{MB}}_t^* - {\mathrm{MC}}_t^* > \mu _t - \varepsilon _t) \\&=F({\mathrm{MB}}_t^* - {\mathrm{MC}}_t^*) \end{aligned} \end{aligned}$$

where *F* is a distribution function. The probability of smoking at time *t* conditional on not having smoked until then can also be written as a hazard function in which the opportunity arrival rate $\psi _t$ is taken into account:

4 $$\begin{aligned} \theta \left( t\right) = \psi _t F({\mathrm{MB}}_t^{*} - {\mathrm{MC}}_t^{*}) \end{aligned}$$

where $\theta \left( t\right)$ is the smoking initiation rate. As ${\mathrm{MB}}_t^* - {\mathrm{MC}}_t^*$ increases or the opportunity arrival rate goes up, the hazard rate of smoking initiation increases, and thus the probability to start smoking increases.

To explain the pattern of smoking initiation from the mid teens to the early twenties one has to assume that the opportunity arrival rate has a peak in this age range or the difference between marginal benefits and marginal costs is positive only in this age interval. It may also be that opportunities are less likely to arrive for early teens or beyond early twenties. Perhaps, peer groups influence smoking behavior such that non-smokers hang-out or are partnered with non-smokers and individuals who have never smoked are less likely to be confronted with an opportunity.

In their rational addiction model, Orphanides and Zervos [37] introduce heterogeneity across individuals on the basis of addictive tendencies. There are non-addicts and potential addicts. Before, they start using an addictive good, individuals are uncertain about their addictive tendency. If an individual is of the addictive type (s)he will experience a negative utility effect related to the detrimental addictive side effects of past consumption. Individuals may fear to be of the addictive type and, therefore, abstain from starting to smoke.^5^ An alternative explanation for the observed age pattern of smoking initiation is that there is heterogeneity in individual behavior such that for some individuals marginal costs are always larger than marginal benefits, and therefore, they abstain from smoking. If for other individuals marginal benefits of smoking initiation are larger than marginal costs in the relevant age range, all these individuals will end up becoming smokers in their teens or early twenties. Then, the observed pattern of smoking initiation is caused by one group of individuals who all start smoking in the relevant age range and another group of individuals who abstain from smoking throughout their life.

In our simple model of smoking initiation it is clear how tobacco control policies may influence decision making of individuals. Tobacco prices will increase marginal costs of smoking initiation and, therefore, reduce the probability that an individual will start smoking at a certain age. Individuals may take future tobacco prices into account as well. If so, this will affect the marginal costs more strongly. The non-price tobacco control policies may affect marginal benefits and/or marginal costs of smoking initiation. All of these policies provide information about the negative health consequences of smoking and, therefore, increase the perceived marginal costs of smoking. In addition to this, smoke-free air laws may reduce marginal benefits of smoking as smoking is no longer possible in certain public domains. Bans on advertising promotion and sponsorship may be beneficial to early teens, since without advertising, they may not be tempted to think that smoking may be fun. Health warnings on tobacco product packaging will increase the perceived costs of smoking but may also reduce the marginal benefits of smoking as the pleasure derived from smoking goes down. Finally, smoking cessation treatment policies, i.e., programs to help dependent smokers stop smoking may be informative to non-smokers about potential addiction and, therefore, not only increase marginal costs but also reduce marginal benefits from smoking initiation.

## Smoking in Europe

### Tobacco control policies and prevalence of smoking

Nowadays, strict regulations on tobacco use are common. Only a few decades ago this was not the case. Up to the 1960s, there were almost no tobacco control policies, neither on smoking in the public domain nor on advertising, as knowledge about the negative health consequences of smoking was limited. In the United States, the 1964 Surgeon General’s report gradually changed the public opinion about tobacco use when it became clear that there are several adverse health consequences related to smoking.

Also in Europe, concerns about negative health consequences of tobacco use started in the 1960s. However, this lead only to some minor regulations in a few countries that advocated smoking cessation and restricted advertisements on tobacco. For a long time, economic interests dominated concerns over health consequences of smoking. The “golden years of tobacco” lasted until the mid-1980s when the European Union (EU) started to implement restrictive tobacco control policies by passing legislation and making tobacco control policies a part of European Union law.

**Table 2:** Information about non-price tobacco control policies: first year in which a policy was implemented Source: Nguyen et al. [34]; see also “Appendix B”

	Smoke-free air	Large direct health warning labels	Comprehensive bans on advertising and promotion	Treatments to help dependent smokers stop
Austria	1994	1974	1994	2001
Germany	1971	1976	1976	1976
Finland	1991	1975	1990	1974
France	2001	1981	1973	1998
Ireland	1994	1990	1970	1991
Italy	1974	1992	1982	1983
Netherlands	1989	1989	1989	1995
Portugal	1982	1990	1982	2001
Spain	1987	1987	1993	1990
Sweden	1993	1973	1993	1969
United Kingdom	2003	1990	1989	1986

Table 2 shows that the first tobacco control policies implemented were mainly bans on advertisement and health warnings on packages. Among the eleven European countries in our empirical analysis, six had already passed some restrictive regulations on tobacco advertisements in 1985, five had done so a few years later. By 1990, almost all countries had meaningful restrictions on advertisement and health warnings on packages. The first country to implement any tobacco control policy was Sweden by passing a law restricting tobacco advertisements in 1969. The last country to implement any tobacco control policy was The Netherlands in 1989. The late introduction may be related to The Netherlands for a long time being the second largest producer of tobacco products and the second largest exporter of cigarettes.

To establish how smoking initiation is affected by tobacco control policies, we use the Tobacco Control Policy Index (TCPI) as an indicator. The TCPI has four main components: smoke-free air laws, comprehensive bans on advertising and promotion, health warnings on tobacco product packaging and treatment to help dependent smokers stop. Nguyen et al. [34] created the TCPI adopting the scoring system introduced by Joossens and Raw [22]. It allocates points to several components of each policy. Smoke-free air laws policy, for example, has four components: Bans in cafes and restaurants, bans in public transport, bans in other public places and bans in workplaces excluding cafes and restaurants. The comprehensive bans on advertising and promotion include bans on radio and television, print media, cinema, sponsorship etc. The health warning labels on tobacco products vary by size, color and pictures. Finally, the treatments to help dependent smokers stop include quit lines and reimbursement of treatment and medications.

The overall index reflects the sum of the scores given to each of the four policies which themselves are sums of the scores given to each of the components (see “Appendix B” for details). Since the same scoring system is used for all countries, the index can be used for cross-country comparisons of the strictness of tobacco control policies. Clearly, the TCP index has its limitations. The way various components are calculated is somewhat arbitrary. Furthermore, the index does not include information about the enforcement of various policies. Finally, two countries can have the same score even though they apply different tobacco control policies. Therefore, in a sensitivity analysis we also investigate the separate effects of each of the four main components.

**Fig. 1:**
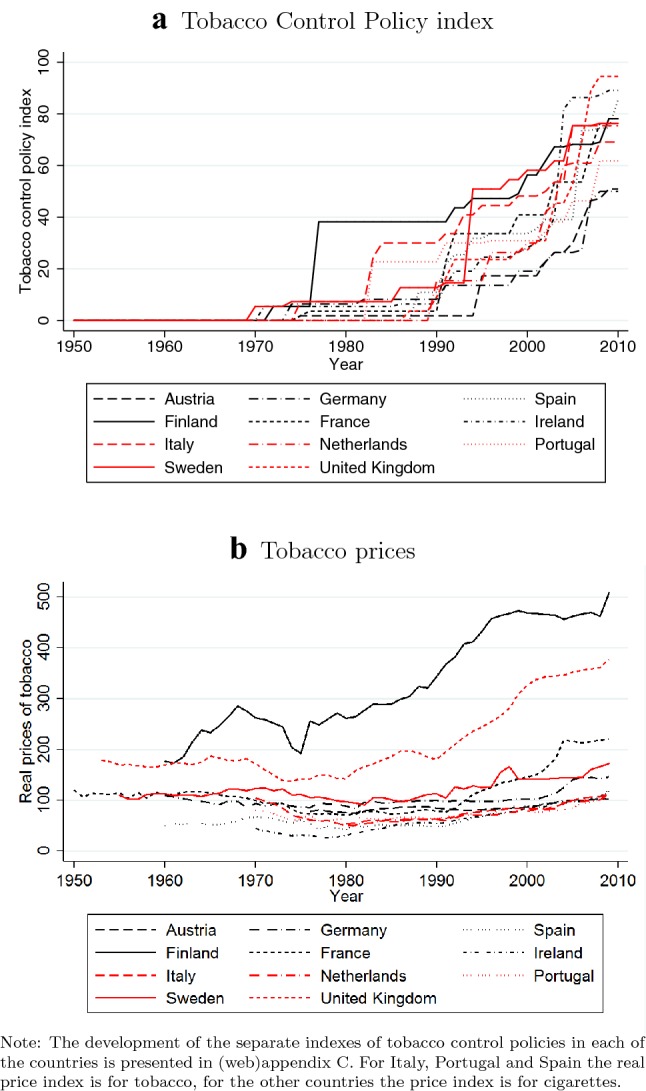
Tobacco Control Policy index and tobacco prices in eleven European countries; 1950–2010

Figure 1a displays the evolution of the TCPI in eleven European countries from 1950 until 2010. Before 1969, the index remains at zero, because there was no tobacco control policy in any of the eleven countries. After 1969 the index slightly increases in many countries, and after 1990 it increases rapidly. There is substantial variation between countries for all tobacco control policies as the first implementation dates and the levels of policy scores differ considerably. Such differences also exits in the tobacco prices. Figure 1b displays the trends in real tobacco prices in eleven countries. Even though real prices of tobacco have been increasing over time in all countries, the increase is much steeper in countries such as Finland and United Kingdom than it is in other countries.

**Fig. 2:**
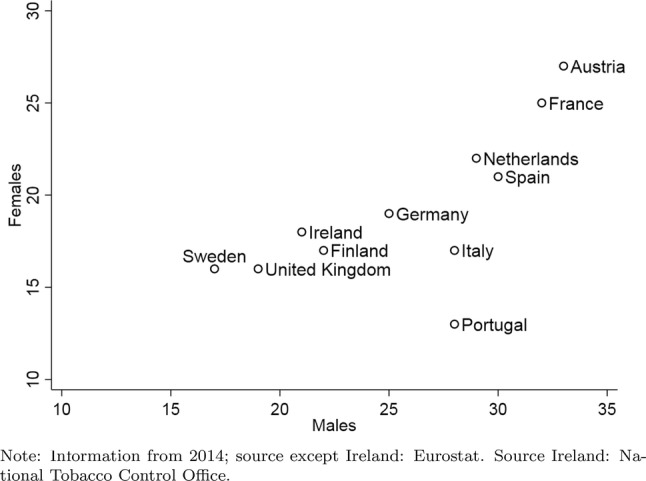
Current smokers (percentage of population over 15 years of age)

Figure 2 provides information about smoking prevalence, i.e., the percentages of current smokers in the population over 15 years of age. There are clear differences between countries and between males and females. For males, the highest smoking prevalence is in Austria, France and Spain, with more than 30% currently smoking. In Sweden and the UK less than 20% of the adult males smoke. For females, the highest smoking prevalence of 25% or more is in Austria and France, the lowest with 13% is in Portugal. In all countries females are less likely to smoke than males although in Sweden the difference is only 1%-point.

### Smoking initiation

To study the uptake of smoking, we use data from a special Eurobarometer (429) covering residents in EU member states aged 15 years and over.^6^ These data collected in November/December 2014 through face-to-face interviews in people’s homes focused on attitudes towards tobacco and electronic cigarettes. The starting point of the survey was the question “Regarding smoking cigarettes, cigars, cigarillos or a pipe, which of the following applies to you?”^7^ If the answer was “currently smoke” or “used to smoke but has stopped”, the following question was “How old were you when you started smoking on a regular basis, i.e., at least once a week?” We use this retrospective information to reconstruct the history of smoking initiation necessary to model the starting rates of tobacco use.

**Table 3:** Cumulative probability to have started smoking by ages of 15, 20, 25 and 30

Age	Females				Males			
	15	20	25	30	15	20	25	30
Austria	4	38	39	39	8	41	43	43
Finland	10	44	47	48	11	40	50	52
France	12	42	52	54	15	52	61	63
Germany	8	39	42	43	12	46	50	51
Ireland	6	34	39	39	7	33	40	41
Italy	3	24	33	35	7	32	37	37
Netherlands	14	42	48	49	16	44	49	54
Portugal	7	31	36	37	8	43	49	50
Spain	12	39	42	42	13	42	47	49
Sweden	7	29	37	38	10	35	40	41
United Kingdom	13	33	38	39	5	37	43	44

*Source*: Authors’ calculation based on a Kaplan–Meier survivor function estimator using Eurobarometer (429) data. The standard error of each estimate is about 3–4 %-point. Therefore, not all cross-country estimates are significantly different from each other. The starting rates and cumulative starting probabilities of smoking in each of the countries is presented in (web)appendix C

The data we use are from eleven European countries for which we have information on tobacco control policies and tobacco prices. In our baseline estimates, we restrict our sample to individuals born from 1970 onward. As Table 3 shows, the age pattern of smoking initiation is very similar across the countries. There is a strong increase between age 15 and 20, a slight increase between age 20 and 25 and a very small increase later on. However, there are big cross-country differences in cumulative starting probabilities by age 30. For males, the share of the population that has ever smoked or is still smoking ranges from 37% in Italy to 54% in The Netherlands. For females it ranges from 35% in Italy to 54% in France.^8^

## Empirical analysis

### Setup empirical analysis

In our empirical analysis, we use a mixed proportional hazard model with a fully flexible baseline specification. Since the underlying dynamics of smoking initiation are expected to be gender-specific, we perform a separate analysis for males and females. In this setup, the duration of stay in the non-smoking state is equivalent to the age of the individual, where we assume that individuals are exposed to possible initiation to smoking from age eleven onward. The starting rate of smoking at time *t* (*t* = 0 at age eleven) of individual *i* in country *j* conditional on observed individual characteristics *x*, tobacco control policy index *B*, real tobacco price *P* and unobserved individual characteristics *u* is defined as

5 $$\begin{aligned} \theta (t\mid x_{i},B_{ijt}, P_{ijt},u_{i})=\lambda _{i} (t) \exp \left( \alpha _j + x_{i}^{\prime }\beta + \delta B_{ijt} + \rho \log (P_{ijt}) + u_{i}\right) \end{aligned}$$

where $\alpha _j$ represents country fixed effects to allow for unobserved time-invariant differences between countries.^9^ Furthermore, $\delta$ measures the effect of non-price tobacco control policies, $\rho$ measures the effect of prices, and $B_{ijt}$ and $P_{ijt}$ are time varying variables which capture within country calendar time variation of smoking restrictions and tobacco prices. Although within a country in a particular year the same smoking restrictions apply to all individuals in that country, the effect of a smoking restriction at a particular age depends on the year of birth of the individual. Therefore, the tobacco control policy at any given age is country-specific and individual-specific. Parameter $\rho$ measures the effect of tobacco prices, where $\log (P_{ijt})$ is the natural logarithm of the real prices of tobacco. Again, the calendar time variation in tobacco control policies is translated into age-specific variation, because a change in tobacco control policy index in a particular year affects individuals from different birth years at a different age. The age variation in the tobacco control policies is country-specific, since there is variation in the years in which these policies were introduced. Identification of the non-price tobacco control policies comes from cross-country variation in the timing of these policies and the cross-individual variation over age when these policies were implemented. In other words, we use a before–after approach in a hazard rate framework, where the before–after variation is caused by the introduction of tobacco control policies in a particular year and in a particular country. This induces an age-specific shift in the starting rate of smoking. The observed characteristics *x* refer to education, birth year and degree of urbanization of the place of residence (see “Appendix A” for details). The vector of parameters $\beta$ represents the effects of other control variables including country-specific birth year trends. Furthermore, $\lambda _i (t)$ represents individual duration dependence. As indicated before, we assume that everyone becomes vulnerable to the risk of initiation into smoking at age eleven. This is because in all of the countries, almost no one starts using tobacco before the age of eleven. Because of this assumption, the duration dependence actually becomes age dependence. Finally, $u_i$ denotes individual unobserved heterogeneity in the starting rates of tobacco use which we model as random effects. They control for differences in time-invariant unobserved susceptibility of individuals to tobacco use. Note that the decision to start smoking often occurs before an individual obtains his or her final educational attainment. Therefore, we assume educational attainment to be an indicator of cognitive ability. By way of sensitivity analysis we also did estimates excluding educational attainment as an observed characteristic, finding similar results.

Duration dependence is specified in a fully flexible way by means of a step function:

6 $$\begin{aligned} \lambda _i(t)=\exp \left( \sum _{k}{\lambda _{ik}I_{k}(t)}\right) \end{aligned}$$

where $k (= 1,\ldots ,11)$ is a subscript for age categories starting from age 11 and $I_{k}(t)$ are time-varying dummy variables that are one in subsequent categories, 10 of which are for individual ages or age intervals (age 11 and $12,\ldots ,$ 17, 18–19, 20–22) and the last interval is for ages above 22 years. Because we estimate a constant term in the analysis, we normalize $\lambda _{i,1}=0$.

The conditional density function of the completed durations until the first use of tobacco can be written as

7 $$\begin{aligned}&f(t\mid x_{i},B_{ijt}, P_{ijt},u_i)=\theta (t\mid x_{i},B_{ijt}, P_{ijt},u_i)\nonumber \\&\quad \exp \left( -\int _{0}^{t}\theta (s\mid x_{i},B_{ijt}, P_{ijt},u_i){\mathrm{d}}s\right) \end{aligned}$$

We integrate out the unobserved heterogeneity such that density function for the duration until tobacco uptake *t* conditional on *x* becomes

8 $$\begin{aligned} f( \mid x_{i},B_{ijt}, P_{ijt})=\int _{u_{i}}f(t\mid x_{i},B_{ijt}, P_{ijt},u_i){\mathrm{d}}G(u_i) \end{aligned}$$

where *G*(*u*) is assumed to be a discrete mixing distribution with 2 points of support $u_{1}$ and $u_{2}$. This reflects the presence of two types of individuals in the hazard rate for tobacco uptake. The associated probabilities are denoted as follows: $Pr (u = u_{1}) = p$ and $Pr (u = u_{2}) = 1-p$, where *p* is modeled using a logit specification, $p=\frac{\exp (\alpha )}{1+\exp (\alpha )}$. Individuals who do not start using tobacco until the time of the survey are considered to have right-censored durations until smoking initiation. The inflow nature of the data guarantees that there are no left censored individuals.

**Table 4:** Baseline parameter estimates mixed proportional hazard model

	Males		Females	
Tobacco control policies	− 0.04	(0.8)	− 0.05	(1.2)
Tobacco prices	− 0.14	(2.8)**	0.05	(0.7)
Education 2	− 0.72	(3.4)**	− 0.63	(3.3)**
Education 3	− 1.84	(8.4)**	− 1.47	(7.2)**
Education 4	− 2.20	(8.5)**	− 1.56	(5.9)**
Small/mid town	0.17	(1.3)	0.32	(2.2)**
Large town	− 0.01	(0.1)	0.29	(2.2)**
Age 12	0.37	(1.1)	0.58	(1.6)
Age 13	1.75	(5.4)**	1.53	(4.4)**
Age 14	2.52	(8.1)**	2.40	(7.2)**
Age 15	3.01	(9.6)**	3.16	(9.5)**
Age 16	3.47	(10.9)**	3.81	(11.5)**
Age 17	3.37	(10.2)**	4.10	(11.9)**
Age 18	3.60	(10.6)**	4.41	(12.2)**
Age 19	3.19	(8.8)**	4.19	(10.6)**
Age 20+	1.27	(3.0)**	2.04	(4.6)**
$u_2$	− 3.19	(11.5)**	− 3.84	(20.6)**
$\alpha$	0.04	(0.3)	− 0.75	(11.1)**
−LogLikelihood	3541.8		3962.9	
Observations	2065		2442	

All estimates contain country fixed effects and country-specific birth year trends; in parentheses absolute t-statistics;

** (*): significant at a 5% (10%) level

### Baseline parameter estimates

Table 4 presents our baseline parameter estimates obtained by the method of Maximum Likelihood. The first column presents the parameter estimates for males, the second column for females.^10^ Panel a of Table 4 contains the baseline parameter estimates in which we focus on the aggregate indicator for tobacco control policies. Parameter estimates of the country fixed effects and the country-specific birth year trends are not reported. Our main parameter of interest, related to the tobacco control policies index is negative but insignificantly different from zero for both males and females. Parameter estimates for tobacco control policies are economically insignificant as well. A complete smoking ban in public transportation services, for example, corresponds to around 0.1% decrease in the smoking initiation hazards.^11^

Tobacco prices have a negative effect for males but not for females. It is not clear why the effect of tobacco prices is gender-specific. However, it is not a surprising result as the same result was found by Cawley et al. [7]. We can only speculate that it is related to the different developments in smoking prevalence. Contrary to the developments for males, in the past decades, smoking of females has increased a lot. It could be that the effect of tobacco prices on smoking initiation is mitigated because of this growth. Because of this, the age of onset of smoking goes up with tobacco prices for males but not for females. As to the personal characteristics, for males and females a lower educational attainment has a positive effect on the smoking initiation rate. Higher education—which we assume to be an indication of ability—has a negative effect on the smoking initiation rate. Females in big cities have a higher starting rate. For males, we do not find any effect for urbanization. The age dependence pattern reflects the age-related fluctuations in the smoking initiation rates. Parameter estimates for unobserved heterogeneity show that for both males and females there is unobserved heterogeneity in the starting to smoke rate. Among the males, 54% has a high starting rate, while 46% has a low starting rate. Among females, these shares are 39 and 61, respectively. The starting rate of the second group is much smaller than the starting rate of the first group. This implies that although some individuals have a positive probability to start smoking, this probability is so small that they will never do that (see Abbring [1] for a discussion on the distinction between ex ante abstainers and ex post abstainers.)

Quite a few of our findings are in line with the results from the previous studies. For example, the negative effect of educational attainment on smoking initiation is a familiar finding. Also, as discussed earlier, the gender-specific differences in the effect of tobacco prices on smoking initiation is a result that has been established before. The finding that tobacco control policies other than tobacco prices for males have no effect on smoking initiation confirms the results of some but contradicts the results of some other studies. As indicated in “Previous studies”, where we reviewed earlier studies on the effect of tobacco control policies there is no common conclusion. Depending on the particular policy sometimes there are even mixed findings within one particular study. Nevertheless, it should be noted that whereas the previous studies often focus on one particular tobacco control policy in one particular country we investigated how overall tobacco control policies affected smoking initiation in a cross-country analysis.

### Sensitivity analysis

The main finding in our baseline estimates is that tobacco prices have a negative effect on smoking initiation of males but have no effect on smoking initiation of females. Furthermore, tobacco control policies do not affect smoking initiation. Neither for males nor for females. Since we want to be sure that these main findings do not depend on peculiarities of our analysis we performed a wide range of sensitivity analysis. There is a variety of potential confounders that might cause a spurious lack of effect for tobacco control policies on smoking initiation other than the negative effect of prices for males. It is possible that ignoring calendar time trends representing a changing attitude toward smoking absorbs the effect of tobacco control policies. Therefore, we investigated whether our results depend the inclusion of a calendar time trend in the hazard rate models. Another possibility is that different control policies have opposite effects on smoking initiation. If so, these effects could cancel out when different policies are combined into one indicator. Therefore, we investigated whether specific tobacco control policies matter. Finally, it is possible that our findings are specific for young birth cohorts or specific countries. Therefore, we investigated whether our findings change if we include older birth cohorts in the analysis. Since these are only available for a subset of countries this sensitivity analysis also provides information about the robustness of our findings when we exclude some countries from the analysis.

As a first check on the robustness of our findings, we estimated the same models with slightly different specifications. Panel a of Table 5 shows how the effect of the TCPI is influenced by changes in the specification of birth year and calendar time trend. In panel a1 the country-specific birth year trends are replaced by a common birth year trend. In panel a2, the country-specific birth year trends are reintroduced in addition to a general calendar time trend. The parameter estimates of the TCPI are not very much affected. For males, replacing the country-specific birth year trends by a common birth year trend changes the sign of the TCPI parameter but this is still insignificantly different from zero. The introduction of a calender time trend is not very important. For females all parameter estimates of the TCPI-index are very much the same, i.e., small and insignificantly different from zero.

To further investigate the robustness of our findings we performed a range of sensitivity analysis. In panel b of Table 5 we report the effects of particular types of tobacco control policies: smoke-free air laws, bans on advertising promotion and sponsorship, health warnings on tobacco product packaging and stop smoking treatment policies. Both for males and females, none of the separate tobacco control policies has an effect on the age of initiation to smoking.

**Table 5:** Parameter estimates effect tobacco control policies; sensitivity analysis

	Males					Females				
	TCP		Prices		−LogL	TCP		Prices		−LogL
*a. Alternative specifications*										
1. General birth year trend	0.06	(1.6)	− 0.13	(1.8)*	3553.0	− 0.02	(0.1)	0.07	(0.2)	3974.5
2. General calendar time trend	− 0.04	(0.7)	− 0.21	(2.8)**	3541.8	− 0.05	(1.2)	0.07	(0.6)	3962.9
*b. Type of TCP*										
1. Smoke-free	− 0.03	(0.8)	− 0.21	(2.8)**	3541.8	− 0.01	(0.5)	0.07	(0.9)	3963.5
2. Advertising	0.01	(0.2)	− 0.20	(2.7)**	3542.1	0.02	(1.2)	0.04	(0.5)	3963.0
3. Health warnings	− 0.03	(0.6)	− 0.20	(2.7)**	3541.9	− 0.01	(1.4)	0.05	(0.7)	3963.2
4. Stop smoking treatment	− 0.05	(1.1)	− 0.19	(2.6)**	3541.5	− 0.05	(1.5)	0.05	(0.7)	3963.3
*c. Additional sensitivity analysis*										
1. Any TCP	0.03	(1.5)	− 0.21	(2.8)**	3541.2	− 0.08	(0.4)	0.05	(0.7)	3963.4
2. Bans in public places	− 0.01	(0.1)	− 0.20	(2.7)**	3542.1	0.02	(0.2)	0.05	(0.6)	3963.4
3. Bans in workplaces	− 0.01	(0.2)	− 0.20	(2.7)**	3542.2	0.02	(1.5)	0.05	(0.6)	3962.5
4. Birth year from 1960	− 0.04	(0.7)	− 0.09	(1.7)*	6028.2	− 0.02	(0.6)	0.05	(0.6)	4734.6
5. Birth year from 1950	− 0.03	(0.2)	− 0.08	(1.4)	4647.4	− 0.04	(1.0)	0.08	(1.1)	4187.8

All estimates contain country fixed effects and (apart from panel a1) country-specific birth year trends as well as the other individual characteristics presented in Table 4. The estimates and panels a, b and c1–c3 are based on 2065 males and 2442 females. In estimation c4, the numbers of observations are 3075 for males and 2815 for females; countries excluded due to the unavailability of the price information are Austria and The Netherlands. In estimation c5, the numbers of observations are 2270 for males and 2297 for females; countries excluded due to the unavailability of the price information are Austria, Ireland, Italy, Portugal and The Netherlands. In parentheses absolute t-statistics

** (*): significant at a 5% (10%) level

Panel c of Table 5 presents further sensitivity outcomes. In panels c1 to c3 the tobacco control policy index is replaced by a dummy variable. In panel c1 the dummy variable is 1 if any tobacco control policy is implemented. In panel c2 it is 1 if smoking is banned in public places, in estimation c3 it is 1 if smoking is banned in workplaces. Therefore, in these estimations, we compare the age of onset of smoking for individuals before and after a tobacco control policy is implemented. The parameter estimates show that our conclusion remains the same. In panels c4 and c5 we also use information on older cohorts, those who were born after 1959 and after 1949, respectively. In some countries the information on tobacco prices is not available for early years, in which case we exclude these countries from this part of the analysis. In panel c4, countries excluded due to the unavailability of the price information are Austria and The Netherlands. In panel c5, Austria, Ireland, Italy, Portugal and The Netherlands are excluded. Although the sample sizes and cohort structure considerably change, our conclusions remain same. This sensitivity analysis also shows that a possible recall bias due to the self-reporting of the data is not an issue. And, it shows that our findings are robust to excluding some countries from the analysis.

## Conclusions

In the past decades, in addition to tobacco price policies, many countries introduced non-price tobacco control policies to reduce smoking. Empirical evidence on the effectiveness of such policies on smoking behavior is inconclusive in the sense that some studies find that tobacco control policies reduce smoking, while other studies find no effect. These differences in findings are partly due to differences in methodology. Many studies use a repeated cross-section type of approach with the incidence of smoking or the intensity of smoking as dependent variables. The effect of a tobacco control policy is analyzed by studying calendar time variation in these smoking variables. Sometimes, studies exploit cross-regional or cross-state differences in the introduction of a tobacco control policy. If an effect is found on the incidence of smoking it is not clear whether this is caused by a decrease in the uptake of smoking or an increase in the quitting from smoking. If an effect is found on the intensity of smoking it is not always clear whether the incidence of smoking is affected as well. There are also a few studies that focus on the dynamics in smoking, studying the effects of tobacco control policies on the uptake of smoking sometimes in combination with the effect on the quitting from smoking.

Our paper contributes to the literature on the relationship between tobacco control policies and smoking initiation. We quantify non-price tobacco control policies using an index. Clearly, such an index has its limitations. Various components are calculated somewhat arbitrary, while the index does not include information about the enforcement of tobacco control policies. Nevertheless, such an index allows for cross-country comparisons and within-country calendar time variation.

We analyze the effects of tobacco control policies on the age of onset of smoking in eleven European countries which implemented different tobacco control policies in different years. We analyze the overall impact of tobacco control policies and study the separate components of these policies, i.e., smoke-free air laws, bans on advertising promotion and sponsorship, health warnings on tobacco product packaging and stop smoking treatment policies. In our empirical analysis, we use mixed proportional hazard rate models to control for observed as well as unobserved factors that can affect smoking initiation. Our model allows a tobacco control policy which is introduced in a particular calendar year to influence the starting rate of smoking through a shift in the smoking initiation rate. The smoking initiation rate is allowed to be country-specific, while we allow for cross-country differences in birth-year trends.

We find that the starting rate of smoking decreases with educational attainment, is age-specific and for males we find that it decreases with tobacco prices. Our main result is that non-price tobacco control policies have no significant effect on the age of onset of smoking, neither for males nor for females. Current tobacco control policies do not seem to discourage young individuals from starting to smoke. The reason for this is not clear. It could be that young non-smokers do not agree with the implemented policies and, therefore, do not adjust their behavior. Our main findings do not imply that current tobacco control policies are all together ineffective in reducing smoking, since they could have reduced the intensity of smoking or stimulated the quit rate from smoking. Nevertheless, our main findings are alarming, because preventing young people from starting to smoke should be as important as trying to reduce their consumption or trying to convince them to quit once they have started. Even postponing smoking initiation to a later age would be helpful as later tobacco initiation will reduce smoking dependence. It is hard to suggest which type of tobacco control policy will successfully reduce smoking initiation. Clearly, youngsters need to be influenced in their decision making at a relatively young age.

### Electronic supplementary material

Below is the link to the electronic supplementary material.

Supplementary material 1 (pdf 4522 KB)

## Appendix A: Details on our data

Definition of variables:

- Education: Measured in terms of age when the respondent left formal education: No education/still studying, up to 15, up to 20, older than 20.
- Cohort effect (age): Age of the respondent in the survey year to capture the cohort effects.
- Urbanization dummies: Rural town/village, small/mid town, large town.

The table below provides descriptives.

**Table:**

	Females			Males		
	Mean	Min	Max	Mean	Min	Max
Birth year-1900	81.4	69	99	82.1	69	99
*Education dummies*						
1. No education/still studying	0.17	0	1	0.19	0	1
2. Up to 15	0.07	0	1	0.06	0	1
3. Up to 20	0.38	0	1	0.37	0	1
4. Older than 20	0.38	0	1	0.38	0	1
*Urbanization dummies*						
Rural town/village	0.26	0	1	0.25	0	1
Small/mid town	0.44	0	1	0.43	0	1
Large town	0.30	0	1	0.32	0	1
Austria	0.12	0	1	0.09	0	1
Germany	0.13	0	1	0.13	0	1
Finland	0.07	0	1	0.07	0	1
France	0.08	0	1	0.08	0	1
Ireland	0.11	0	1	0.09	0	1
Italy	0.09	0	1	0.10	0	1
Netherlands	0.07	0	1	0.07	0	1
Portugal	0.10	0	1	0.08	0	1
Spain	0.09	0	1	0.09	0	1
Sweden	0.05	0	1	0.11	0	1
United Kingdom	0.08	0	1	0.09	0	1

Based on 2065 males and 2442 females

## Appendix B: The tobacco control policy index

**Table:**

		Max points	Rebased
1	Smoke-free air	22	40
A.	Cafes and restaurants	8	14.6
B.	Public transport	2	3.6
C.	Other public places	2	3.6
D.	Workplaces excluding cafes and restaurants	10	18.2
2	Comprehensive bans on advertising and promotion	13	23.6
A.	Complete ban on tobacco advertising on television	3	5.4
B.	Complete ban on outdoor advertising (e.g. posters)	2	3.6
C.	Complete ban on advertising in print media (e.g. newspapers and magazines)	2	3.6
D.	Complete ban on indirect advertising (e.g. cigarette branded clothes, etc)	2	3.6
E.	Ban on point of sale advertising	1	1.8
F.	Ban on cinema advertising	1	1.8
G.	Ban on sponsorship	1	1.8
H.	Ban on internet advertising	0.5	0.9
I.	Ban on radio advertising	0.5	0.9
3	Large direct health warning labels	10	18.2
A.	Rotating health warnings	2	3.6
B.	Size of warning	4	7.3
C.	Contrasting color (e.g. black lettering on white background)	1	1.8
D.	A picture or graphic image	3	5.5
4	Treatment to help dependent smokers stop	10	18.2
A.	Quit-line	2	3.6
B.	Network of smoking cessation support and reimbursement of treatment	6	10.9
C.	Reimbursement of medications	2	3.6
	Total score	55	100

*Source*: Nguyen et al. [34]

## Footnotes

1 Nagelhout et al. [32] find mixed evidence for this. In England and Ireland smoke-free legislation may have stimulated quits from smoking, while in The Netherlands, this does not seemed to have been the case.

2 Wilson et al. [47] provide an overview of 84 studies on the relationship between Tobacco Control Policies and smoking dynamics and prevalence of smoking. There are only a few studies on smoking initiation. Their main finding is that there is no clear relationship between smoking bans in public places and initiation into smoking.

3 This is similar to job finding theory, where unemployed workers receive a stream of random wage offers from a distribution with certain characteristics. Conditional on the arrival rate of job offers, search costs and the wage distribution the individual decides which wage offers to accept. Of course there are many differences as well. Unemployed search for opportunities to make a transition from unemployment to work, while non-smoking individuals may not be interested per se in a transition to becoming a smoker.

4 Once an individual has started smoking, decisions to keep smoking later on depend on the stock of addictive capital based on the number of cigarettes smoked. Equation (1) is about smoking initiation, so the stock of addictive capital is zero. Darden [12] presents a dynamic model of lifetime smoking behavior focusing on the response of smokers to updated information on their health. Odermatt and Stutzer [36] suggest that a behavioral approach to tobacco control policies may be relevant. Using data from 22 European countries, they relate tobacco control policies to life satisfaction finding no significant effects. Only for smokers who would prefer to stop smoking permanently there is a beneficial effects of smoking bans, a finding which the authors relate to the possibility that smoking bans serve as a self-control device.

5 Other individuals may start smoking, find out that they are of the addictive type and then regret their original decision. Regret may also have a different origin. Suranovic et al. [42], for example, assume bounded rationality such that individuals focus on decisions about current smoking rather than taking future smoking into account. Then, it is possible that later in life individuals regret decisions they made when they were young.

6 Bosdriesz et al. [6] uses 2006, 2009 and 2012 Eurobarometer data from 27 European countries to study the effect of tobacco control policies and quit ratio defined as the ratio of the number of former smokers and the sum of the numbers of former and current smokers. The tobacco control policies are specified on a scale from 0 to 100 based on expert opinions on six domains: tobacco price, smoke-free areas, tobacco control budget, bans on tobacco advertising, health warning labels and support to stop smoking (cessation programs). Bosdriesz et al. [6] find an association between their measure of tobacco control policies and smoking cessation for higher educated individuals but not for individuals with lower and intermediate education.

7 To this question was added that “Smoking cigarettes does not include use of electronic cigarettes.”

8 Note that, as indicated in the footnote to Table 3, we used a Kaplan-Meier survivor function estimator to calculate the cumulative probabilities. The standard error of each estimate is about 3-4 %-point. Therefore, not all cross-country estimates are significantly different from each other.

9 DeCicca et al. [13] find that once they include state fixed effects in their hazard rate analysis, the significant effect of tobacco taxes on initiation into smoking disappears.

10 We also did a joint estimate pooling males and females and introducing a dummy variable for females. The results show that conditional on having the same parameters for males and females, females have a lower starting rate for smoking. However, the Likelihood Ratio statistic for comparing the separate estimates with the joint estimate is equal to 74.0 which is significant at a 5%-level (critical $\chi ^2$-value with 40 degrees of freedom is 55.8). Therefore, we proceed with the separate estimates for males and females.

11 In TCPI indices a smoking ban in public transportation services corresponds to a score of 2.2 out of 100.
