# Supplementary material for: The impact of tobacco control policies on smoking initiation in eleven European countries
Source: Eur J Health Econ. 2019 Aug 9;20(9):1287–301. doi: 10.1007/s10198-019-01090-x (PMC6856042; doi:10.1007/s10198-019-01090-x)

# Web-Appendix C: Country-specific graphs

Figure 1: Separate indexes of tobacco control policies in eleven European countries; 1950-2010

Austria

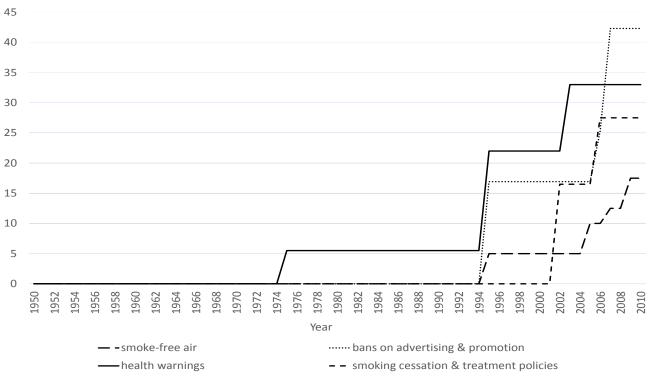


Finland

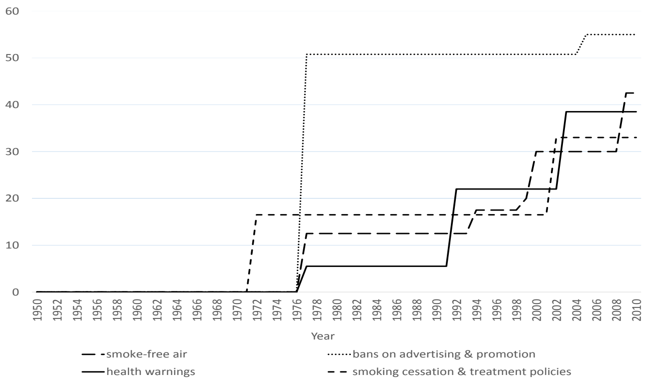


France

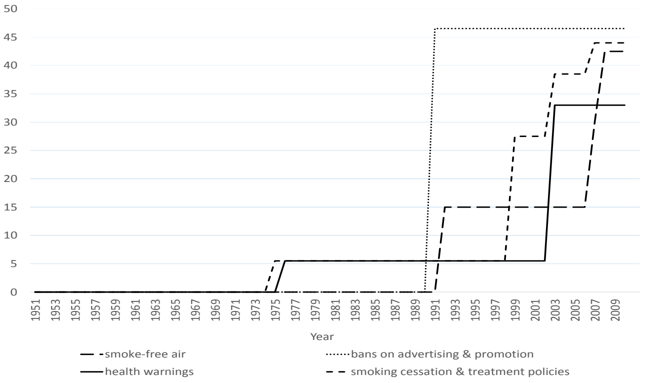


Germany

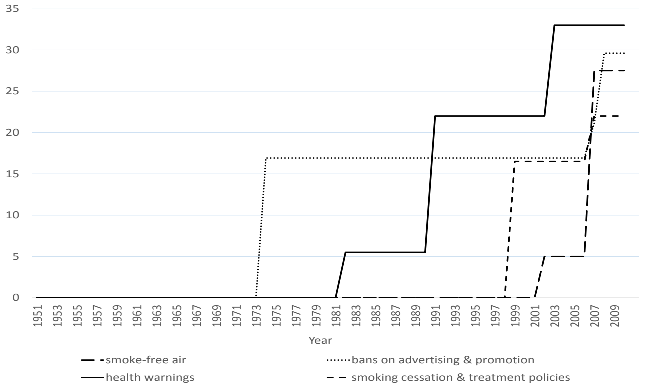


Ireland

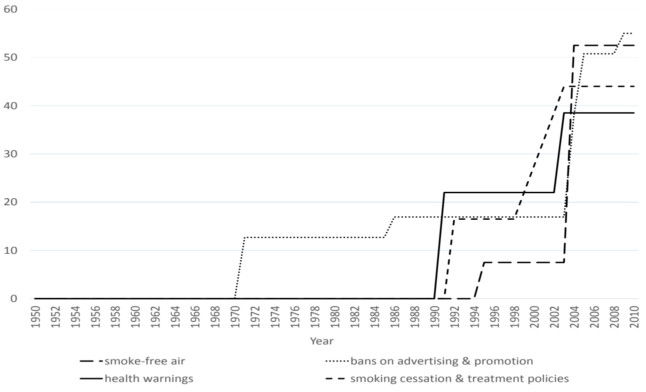


Italy

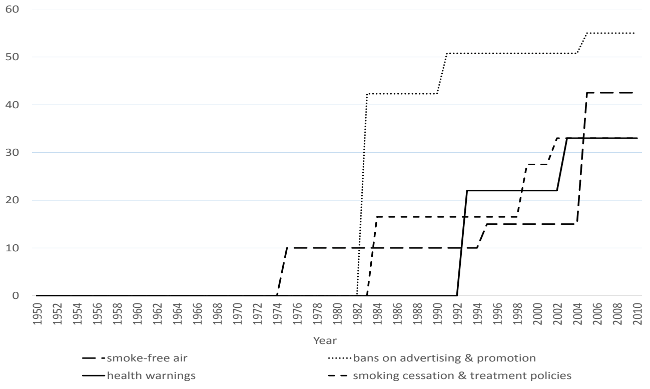

Figure 1 continued.

## Netherlands

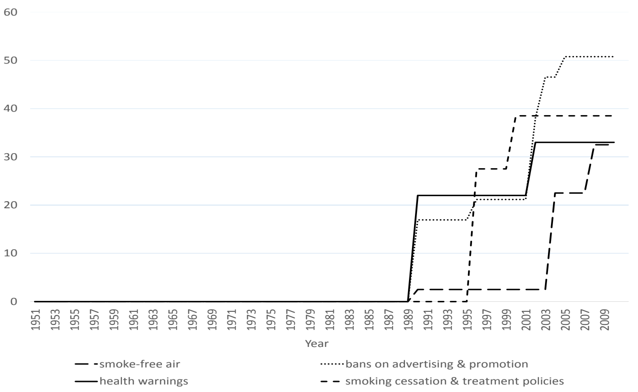


## Portugal

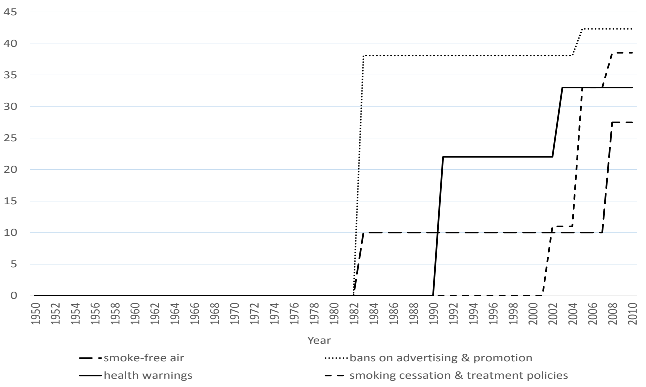


## Spain

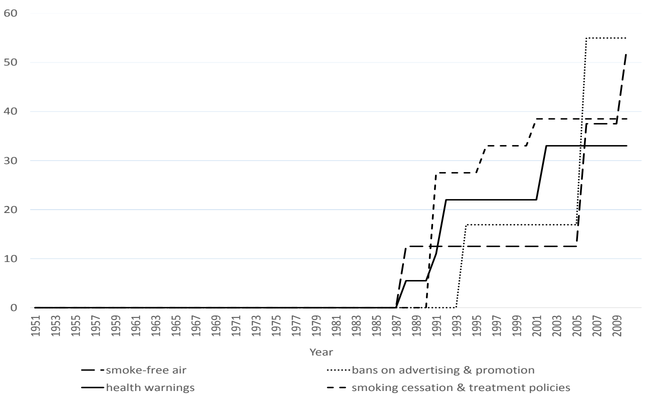


## Sweden

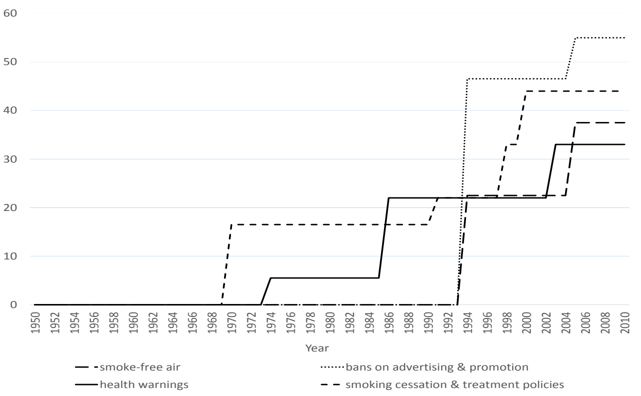


## United Kingdom

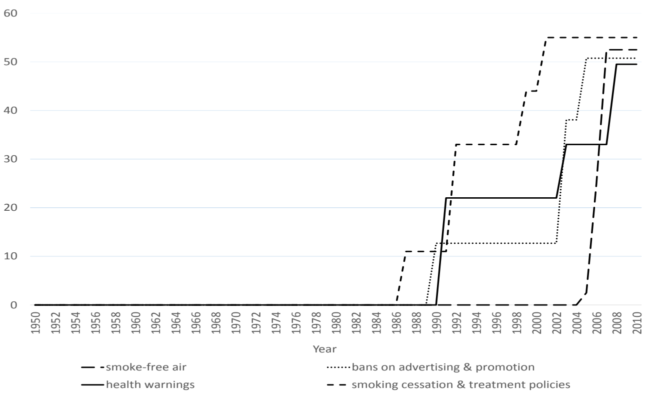

Figure 2: Starting rates and cumulative starting probability of smoking.

## Austria

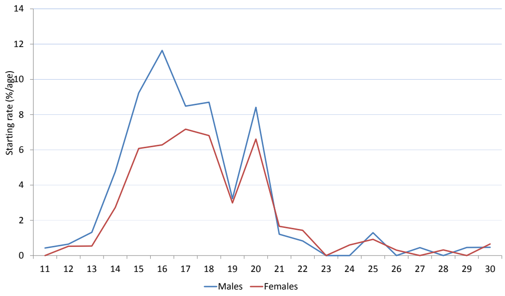


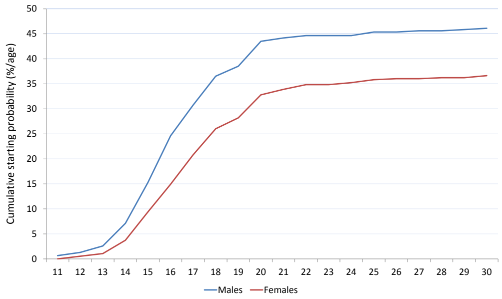


## Germany

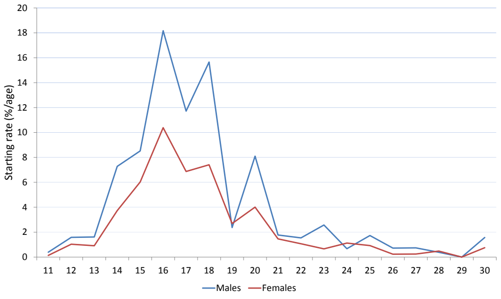


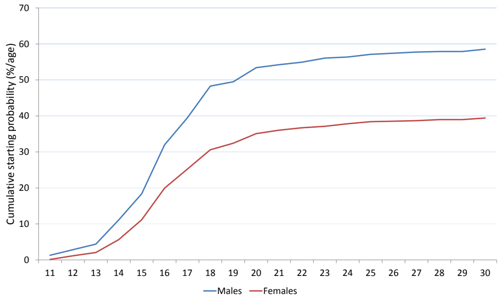


## Spain

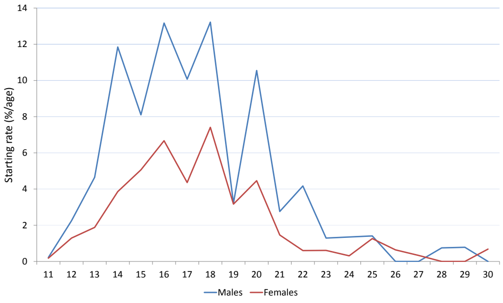


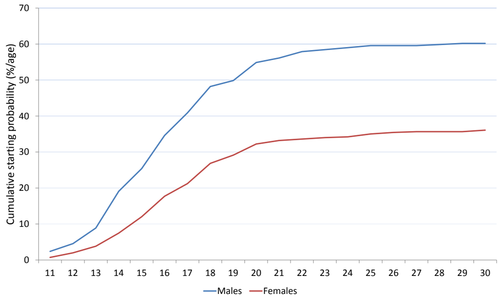


## Finland

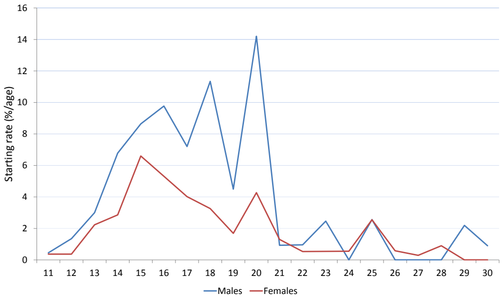


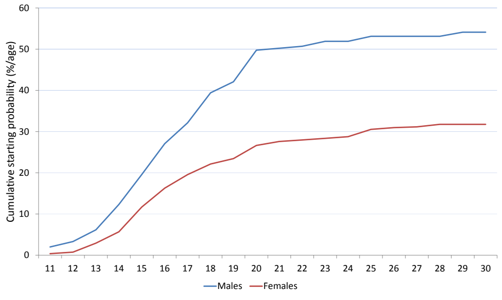

Figure 2 continued.

## France

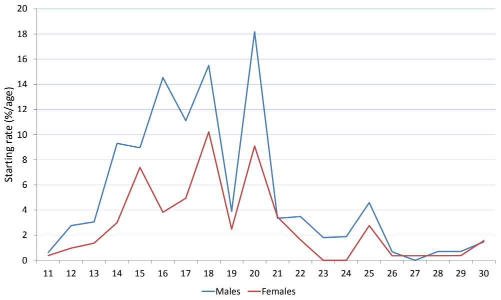


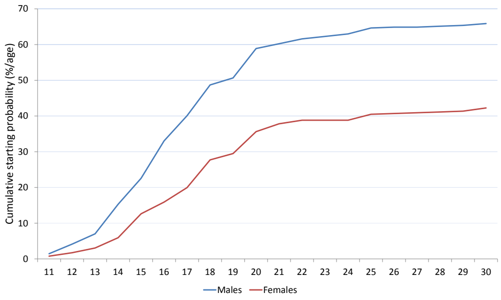


## Ireland

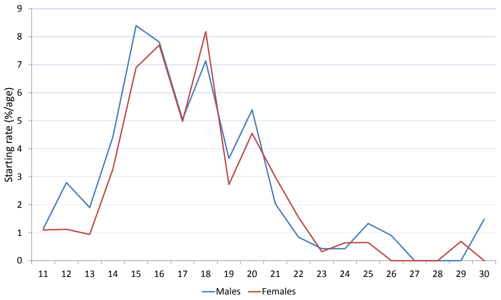


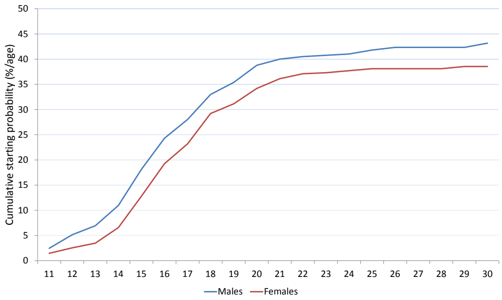


## Italy

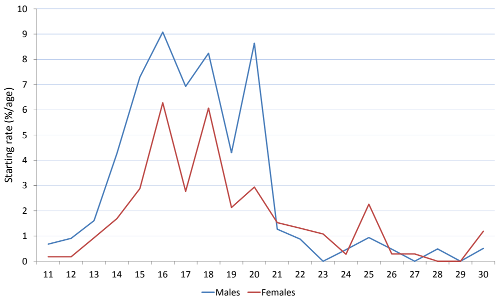


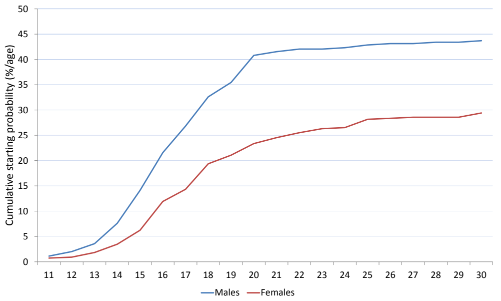


## Netherlands

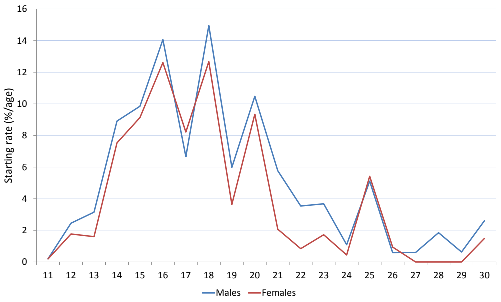


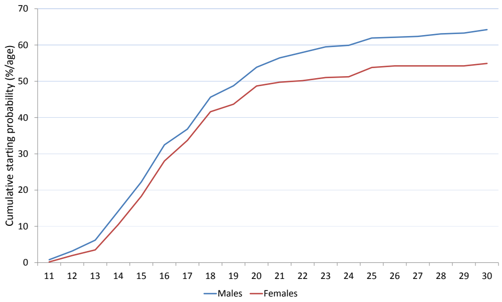

Figure 2 continued.

## Portugal

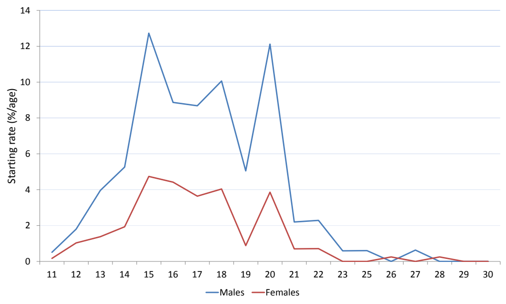


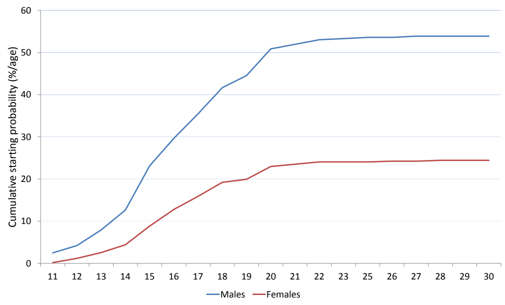


## Sweden

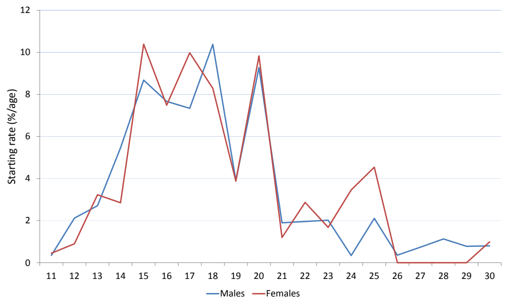


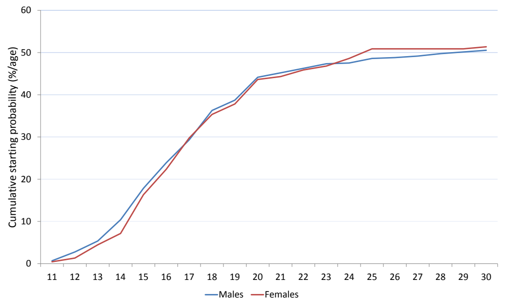


## United Kingdom

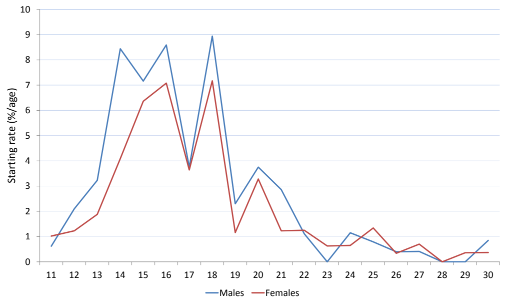


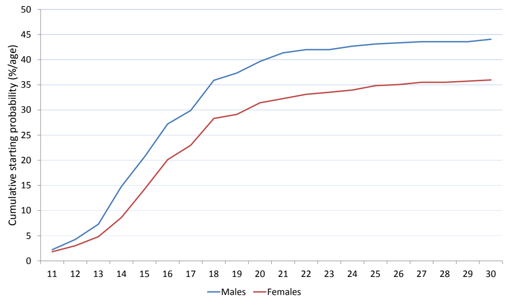

Supplement: Supplementary file 1 — Supplementary material 1 (pdf 4522 KB) [file 10198_2019_1090_MOESM1_ESM.pdf]
